# Characterisation of the Immunomodulatory Effects of Meningococcal Opa Proteins on Human Peripheral Blood Mononuclear Cells and CD4^+^ T Cells

**DOI:** 10.1371/journal.pone.0154153

**Published:** 2016-04-25

**Authors:** Claire Jones, Manish Sadarangani, Susan Lewis, Isabelle Payne, Muhammad Saleem, Jeremy P. Derrick, Andrew J. Pollard

**Affiliations:** 1 Oxford Vaccine Group, Department of Paediatrics, University of Oxford and NIHR Biomedical Research Centre, Oxford University Hospitals NHS Trust, Oxford, United Kingdom; 2 Faculty of Life Sciences, University of Manchester, Manchester, United Kingdom; Universidad Nacional de la Plata, ARGENTINA

## Abstract

Opa proteins are major surface-expressed proteins located in the *Neisseria meningitidis* outer membrane, and are potential meningococcal vaccine candidates. Although Opa proteins elicit high levels of bactericidal antibodies following immunisation in mice, progress towards human clinical trials has been delayed due to previous findings that Opa inhibits T cell proliferation in some *in vitro* assays. However, results from previous studies are conflicting, with different Opa preparations and culture conditions being used. We investigated the effects of various Opa+ and Opa- antigens from *N*. *meningitidis* strain H44/76 in a range of *in vitro* conditions using peripheral blood mononuclear cells (PBMCs) and purified CD4^+^ T cells, measuring T cell proliferation by CFSE dilution using flow cytometry. Wild type recombinant and liposomal Opa proteins inhibited CD4^+^ T cell proliferation after stimulation with IL-2, anti-CD3 and anti-CD28, and these effects were reduced by mutation of the CEACAM1-binding region of Opa. These effects were not observed in culture with *ex vivo* PBMCs. Opa+ and Opa- OMVs did not consistently exert a stimulatory or inhibitory effect across different culture conditions. These data do not support a hypothesis that Opa proteins would be inhibitory to T cells if given as a vaccine component, and T cell immune responses to OMV vaccines are unlikely to be significantly affected by the presence of Opa proteins.

## Introduction

*Neisseria meningitidis* causes approximately 500,000 cases of meningitis and septicaemia worldwide annually, with a case-fatality rate of approximately 10% [1]. Most disease is caused by capsular group A, B, C, W, X and Y organisms. Protein-polysaccharide conjugate vaccines are in routine use globally for capsular groups A, C, W and Y, and group B is the major cause of disease in most temperate countries [2–6]. The Opacity-associated (Opa) adhesin proteins are major phase-variable proteins found in the outer membrane of *N*. *meningitidis*. Each bacterium contains four *opa* genes (*opaA*, *opaB*, *opaD* and *opaJ*), which may encode identical or different Opa proteins, and can therefore express up to four different Opa variants at any one time [7–9]. Opa proteins are critical in meningococcal pathogenesis, mediating bacterial adherence to the nasopharynx and modulating human cellular immunity via interactions with T cells and neutrophils [10, 11]. They are also important during infection with the related species *N*. *gonorrhoeae*, mediating persistence of bacteria in the genitourinary tract via the avoidance of an effective immune response [12].

*N*. *meningitidis* can persist in the human nasopharynx without causing symptoms for several months, and *N*. *gonorrhoeae* can cause prolonged mucosal infection of the genito-urinary tract. This ability to persist relies on their adaptability to the host and their capacity to evade the immune system. Carcinoembryonic antigen-related cell adhesion molecules (CEACAMs) are cell surface glycoproteins found on a range of cell types. Binding of these proteins by various ligands can result in up- or down-regulation of intracellular signalling pathways [12]. Opa protein binding to CEACAMs on the surface of host cells confers the ability to associate with human epithelial, endothelial and leucocytic cells encountered during neisserial infection, indicating a direct effect on the immune response [13]. Although Opa proteins are able to bind to a number of different CEACAMs, CEACAM1 has a broad expression distribution in normal tissues and is the only member of the family present on the surface of T cells. The response of T cells, and particularly CD4^+^ T cells, is important during infection with pathogenic Neisseria as these cells are involved in directing the magnitude and quality of humoral immune response. Antibodies directed against surface structures of *N*. *meningitidis* are important in immunity but gonococci do not induce a strong, protective antibody response following infection [14]. T cells are also important in the generation of immunological memory and possibly cell-mediated immunity, which is therefore relevant to vaccine development [15]. The interaction between meningococci and human T cells and the particular role of Opa proteins in this interaction has therefore been the subject of intense, and conflicting, study in the last decades [16–24]. Furthermore, Opa proteins have been suggested as potential meningococcal vaccine candidates as they elicit high levels of bactericidal antibodies in mice [13]. However, sequence variability of some of the surface-exposed loops and uncertainty regarding their immunomodulatory effect on human T cells has delayed further development into clinical trials.

In this study we investigated the effects of recombinant and liposomal Opa proteins, in addition to Opa+ and Opa- outer membrane vesicles (OMVs) and bacteria based on isogenic strains, on the immunomodulatory interaction between *N*. *meningitidis* and human peripheral blood mononuclear cells (PBMCs) and CD4^+^ T cells. In an attempt to clarify the effects of Opa proteins on CD4^+^ T cells, a number of assays were undertaken using different cell culture conditions, and a variety of Opa+ and Opa- antigens.

## Materials and Methods

### Study subjects

Written informed consent was obtained from 46 healthy adult volunteers recruited to the study (aged 18 to 66 years) prior to collection of a single blood sample. Anyone with a history of previous IMD, a known immunodeficiency, or who was enrolled in another study which may affect their immune responses was excluded. The study was approved by the Oxfordshire C Research Ethics Committee (REC No: 07/H0606/84; UKCRN ID 4609).

### Isolation of peripheral blood mononuclear cells and purification of CD4^+^ T cells

A maximum of 40 ml of blood was collected from each study participant, and heparinised blood (1000 units/ml heparin) was diluted in an equal volume of culture medium buffer (RPMI-1640 medium, HEPES modification, 25 mM HEPES, 50 units/ml penicillin, 50 μg/ml streptomycin, 2 mM L-glutamine [Sigma-Aldrich, Gillingham, UK]). PBMCs were isolated by density gradient centrifugation (Lymphoprep, Axis-Shield, Dundee, UK). Cells were subsequently either labelled with carboxyfluorescein succinimidyl ester (CFSE) prior to culture and stimulation with antigen, or directly processed for purification of CD4^+^ T cells. CD4^+^ T cells were purified from PBMCs by negative selection according to manufacturer’s instructions (CD4^+^ T cell isolation kit II, Miltenyi Biotec, Woking, UK). Following separation, cells were labelled with CFSE prior to culture and stimulation with antigen.

### Cell labelling with carboxyfluorescein succinimidyl ester (CFSE)

To assess proliferation by flow cytometry, fluorescent intracellular labelling of PBMCs and purified CD4^+^ T cells with CFSE was performed prior to culture and stimulation with antigen [25]. In brief, the cells were resuspended in CFSE buffer (PBS containing 2mM EDTA and 0.5% newborn calf serum [NBCS]), and diluted to a concentration of 1 x 10^6^ cells/ml in CFSE buffer containing 2 μM CFSE. Cells were incubated at 37°C with 5% CO_2_ for 10 minutes and washed twice in culture complete medium (containing 10% NBCS). Cell suspensions (2 x 10^6^ cells/ml for PBMCs and 1 x 10^6^ cells/ml for purified CD4^+^ T cells) were prepared with or without IL-2 (final concentration of 1,000 units/ml) (Roche, Welwyn Garden City, UK).

### Cell culture and T cell stimulation

Cells were cultured in flat-bottomed 96-well tissue culture plates (Thermo Scientific Nunc, Loughborough, UK) at a cell density of 2 x 10^5^ or 1 x 10^5^ cells per well for PBMC and CD4^+^ T cells, respectively, in complete culture medium (100 μl per well). To induce the expression of CEACAM1, cells were pre-incubated with IL-2 (1,000 units/ml) for 48 hours at 37°C with 5% CO_2_ prior to antigen addition. For *ex vivo* studies, antigens were diluted prior to addition to the culture plate with or without T cell stimulation using purified anti-human CD3 (UCHT1) and anti-human CD28 (CD28.2) antibodies for a further 96 hours (Table 1). Per participant, each antigen and assay controls were tested in triplicate.

**Table 1:** Antigens used for T cell proliferation assays.

Antigen	Stock concentration	Final concentration in assay
PHA (Sigma-Aldrich)	5 mg/ml	5 μg/ml
Anti-human CD3 + anti-human CD28 antibodies (BioLegend)	1.mg/ml‡	1 μg/ml‡
Isotype control antibody (DAKO)	15 g/ml	25 μg/ml
Polyclonal anti-CEACAM antibody (DAKO)	2 mg/ml	25 μg/ml
OMVs	Variable	50 μg/ml
Inactivated bacteria§	Variable	ratio of 500 bacteria: 1 cell
Recombinant proteins	Variable	1 μg/ml
Liposome proteins	Variable	1 μg/ml

All antigens were diluted in to the required concentration in a buffer containing RPMI-1640 medium, HEPES modification, 25 mM HEPES, 50 units/ml penicillin, 50 μg/ml streptomycin, 2 mM L-glutamine supplemented with 10% heat-inactivated newborn calf serum [Sigma-Aldrich, Gillingham, UK].

PHA = phytohaemagglutinin; SEB = Staphylococcal enterotoxin B; OMVs = outer membrane vesicles;

^‡^concentration per antibody;

^§^Bacteria were washed 3 times in complete medium prior to use;

¶1 x 10^8^ CFU per well for assays with PBMCs and 5 x 10^7^ CFU per well for assays with purified CD4^+^ T cells.

In order to comprehensively investigate the effects of Opa proteins on CD4^+^ T cell proliferation, different Opa+ or Opa- antigens were tested using a combination of culture conditions. The following culture conditions were tested: (a) *ex vivo* culture of PBMCs with antigen for 5 days; (b) PBMCs cultured for 4 days with antigen and IL-2 (following 48 hours pre-incubation with IL-2); (c) PBMCs cultured for 4 days with antigen, IL-2, anti-CD3 and anti-CD28 (following 48 hours pre-incubation with IL-2); (d) purified CD4^+^ T cells cultured for 4 days with antigen, IL-2, anti-CD3 and anti-CD28, (following 48 hours pre-incubation with IL-2). Priority was given to assays involving *ex vivo* PBMCs, as the convention used to assess T cell stimulation and of most interest to vaccine development. Co-ligation culture conditions including anti-CD3 and anti-CD28 were performed as a priority over those with IL-2 alone when the number of cells recovered from blood was insufficient for all assays.

### Antigens for T cell stimulation

An extensive antigen panel consisting of purified recombinant Opa proteins, liposomes, OMVs and killed bacteria from isogenic strains constructed from parent strain H44/76 were studied (Table 1). The isogenic strains used in this study included the wild-type parent strain H44/76, an Opa- strain (M014), a strain expressing OpaD only (M002), and a strain expressing both OpaA and OpaD (M001) [26]. *opa* genes associated with hyperinvasive serogroup B meningococci were cloned, expressed in *Escherichia coli* BL21 (DE3), purified from inclusion bodies using affinity chromatography [13] and incorporated into liposomes [27]. Non-CEACAM binding mutated forms of OpaA and OpaD were constructed by site directed mutagenesis, replacing Gly-172, Ile-174 and Gln-176 with alanine, as previously described [28]. Phytohaemagglutinin (PHA) [29, 30] and Staphylococcal enterotoxin B (SEB) were used as positive controls for the assays. A rabbit polyclonal anti-CEACAM antibody (DAKO, Ely, UK) was used as an inhibitory control for T cell responses while an isotype matched antibody control was used as the comparator [31].

### Analysis of cells using flow cytometry

Generations of proliferating CD4^+^ T cells were assessed by flow cytometry [25]. CD4^+^ T cells were positively identified by staining with a mouse anti-human CD4-APC conjugate (BD Pharmingen, Oxford UK) and dead cells were excluded from the analysis using 7-aminoactinomycin D (7-AAD) (Sigma-Aldrich, UK). Flow cytometry was performed using the FACSCalibur (Becton Dickinson, UK). Data from 10,000 CD4^high^ 7-AAD^low^ events were collected for each sample. Data were collected and stored using CellQuestPro for Macintosh (Becton Dickinson). Assay controls for each participant included unstained (minimum auto-fluorescence) and CFSE-stained unstimulated controls (media only control). For analysis, a histogram plot was created and a marker was used to identify the fluorescence peak of the media only control (maximum fluorescence of the undivided cells). The average number of proliferating events in the final culture population for triplicate measurements was determined. Cell proliferation is defined as a percentage of the total number of CD4^+^ T cells analysed.

### Bactericidal antibody responses following immunisation of mice

Groups of ten 6–7 week old female BALB/c mice (Charles River, Margate, UK) were immunised subcutaneously with 0.2ml of recombinant Opa protein on days 0, 21 and 35 (5 μg per dose). The adjuvant used was the oil-in-water emulsion Sigma Adjuvant System (Sigma-Aldrich), which was prepared according to the manufacturer’s instructions but in a total volume of 2.4ml of antigen solution per vial, and was reconstituted with the antigen on the day of immunisation. Blood was collected by cardiac puncture on day 42 and serum separated by centrifugation at 16,100 x *g* for 10 minutes. Bactericidal activity in mouse serum pooled within each immunisation group was quantified by SBA [32]. Briefly, pooled murine sera was heated at 56°C for 30 minutes to deactivate endogenous complement and then diluted to give a range from 1:4 to 1:1024. Diluted sera were incubated with mid-log phase meningococci (125 cfu) and baby rabbit complement (lot number 11330, PelFreez Biologicals, Rogers, AR) at a final concentration of 25% (v/v). Reactions were incubated for 60 minutes at 37°C in a humidified 5% CO_2_ atmosphere. A sample of each reaction was spread by tilting onto BHI agar plates, which were incubated overnight. The bactericidal antibody titre was reported as the reciprocal of the highest serum dilution at which 50% bacterial survival was observed. Each serum sample was analysed in duplicate against each target strain.

### Statistical analysis

CD4^+^ T cell proliferation was calculated for each antigen as the mean of the triplicate wells, by subtracting the mean proliferation of control wells containing media only. Opa+ antigens were compared to Opa- antigens in the same way. For wild-type and mutant forms of recombinant or liposomal Opa proteins, the mutated forms were considered Opa- in these analyses. Statistical significance was calculated using a paired two-tailed Student's t-test to compare cells from the same donor, with a *p*-value of <0.01 considered as statistically significant. Analysis was carried out using R (version 3.1.2).

## Results

### PBMCs ex vivo CD4^+^ T cell proliferation

**Fig 1:**
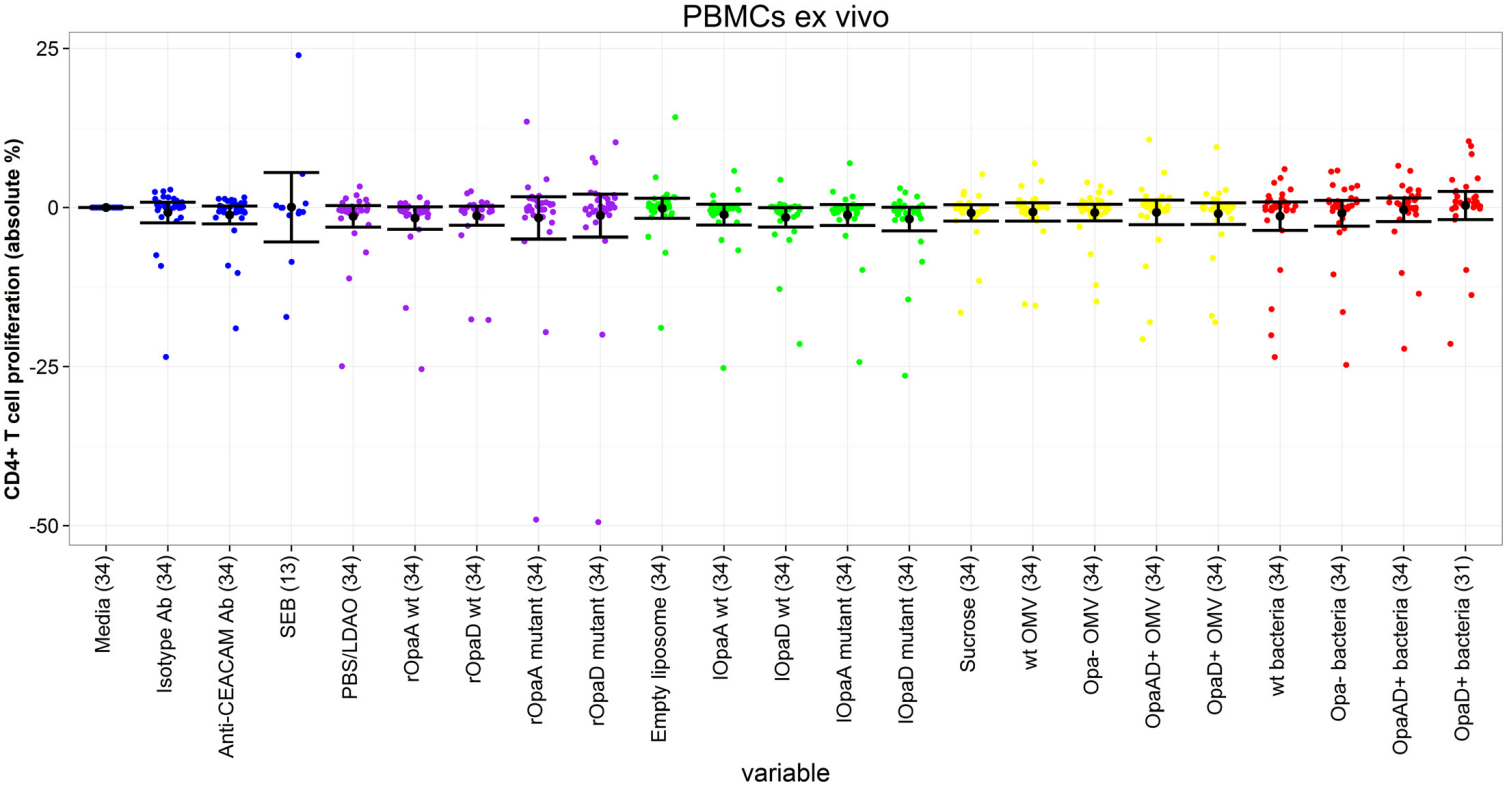
CD4^+^ T cell proliferation following incubation of PBMCs ex vivo with different antigens. Proliferation of each antigen relative to media only control. Each coloured dot represents the value for a single individual (control antigens—blue; recombinant proteins—purple, liposomal proteins—green, outer membrane vesicles—yellow, inactivated bacteria—red). Black dots represent mean value for each antigen, with 95% confidence intervals shown. Number in parentheses after each antigen represents number on individuals in that group. CEACAM = carcinoembryonic antigen-related cell adhesion molecule; SEB = Staphylococcal enterotoxin B; PBS = phosphate-buffered saline; LDAO = llauryldimethylamine-oxide; wt = wild-type. PHA (phytohaemagglutinin) result not shown for clarity, to confine y-axis to relevant range for other antigens.

Proliferative responses to all antigens were low in these conditions, with a maximum amplitude of mean stimulation/inhibition of 1.8%, with the exception of PHA (Table 2, Fig 1). There was statistically significant inhibition of T cell proliferation by the liposomal wild-type OpaD, although the magnitude of this inhibition was low (-1.6%), compared to the stimulatory effect seen with the PHA control (+61.3%). Otherwise there was no significant stimulation or inhibition by any of the Opa+ or Opa- antigens. When Opa+ antigens were compared to their Opa- counterparts there were no significant differences, with the exception that Opa+ bacteria were relatively stimulatory. The magnitudes of these differences were small, +0.5% and +1.3% for OpaA+ OpaD+ and OpaD+ strains respectively. Although culture of PBMCs *ex vivo* without IL-2 and/or stimulation of the T cell receptor (e.g. with PHA or anti-CD3) is a standard method for assessing antigen-specific responses following a vaccination, proliferative responses measured *in vitro* in the absence of such a recent stimulus can be more difficult to detect [33]. Further experiments were therefore performed with additional IL-2 to amplify proliferative responses [34].

**Table 2:** CD4^+^ T cell proliferation following incubation of PBMCs ex vivo with different antigens.

Antigen	Mean % CD4^+^ T-cell proliferation				
	n	vs. media (95% CI)	*p*-value	vs. Opa- (95% CI)	*p*-value
**Control antigens**					
PHA	33	**+61.3 (50.8, 71.8)**	**<0.0001**	-	-
Isotype antibody	33	-0.8 (-2.4, 0.8)	0.3281	-	-
Anti-CEACAM antibody	33	-1.2 (-2.6, 0.2)	0.0979	-	-
SEB	12	+0.1 (-5.4, 5.5)	0.9776	-	-
**Recombinant proteins**					
Protein buffer (PBS/LDAO)	33	-1.4 (-3.1, 0.3)	0.1037	-	-
rOpaA wt	33	-1.7 (-3.4, 0.1)	0.0638	0.0 (-3.3, 3.2)	0.9804
rOpaD wt	33	-1.3 (-2.8, 0.2)	0.0892	0.0 (-3.4, 3.3)	0.9869
rOpaA mutant	33	-1.6 (-4.9, 1.7)	0.3252	REF	-
rOpaD mutant	33	-1.3 (-4.6, 2.1)	0.4501	REF	-
**Liposomal proteins**					
Empty liposome	33	-0.1 (-1.7, 1.5)	0.8741	-	-
lOpaA wt	33	-1.1 (-2.8, 0.5)	0.1719	+0.1 (-0.6, 0.7)	0.8583
lOpaD wt	33	-1.6 (-3.1, 0)	0.0468	+0.3 (-0.4, 0.9)	0.3885
lOpaA mutant	33	-1.2 (-2.8, 0.5)	0.153	REF	-
lOpaD mutant	33	-1.8 (-3.7, 0)	0.0543	REF	-
**Outer membrane vesicles (OMVs)**					
OMV buffer (3% sucrose)	33	-0.9 (-2.1, 0.4)	0.1876	-	-
wt OMV	33	-0.7 (-2.1, 0.7)	0.3308	+0.1 (-0.4, 0.6)	0.6926
Opa- OMV	33	-0.8 (-2.1, 0.5)	0.2213	REF	-
OpaA+ OpaD+ OMV	33	-0.8 (-2.7, 1.2)	0.4234	0.0 (-0.7, 0.8)	0.9393
OpaD+ OMV	33	-1 (-2.7, 0.7)	0.2556	-0.2 (-0.7, 0.4)	0.5561
**Inactivated bacteria**					
wt H44/76	33	-1.4 (-3.6, 0.9)	0.223	-0.5 (-1.5, 0.6)	0.3757
Opa-	33	-0.9 (-2.9, 1.1)	0.3695	REF	-
OpaA+ OpaD+	33	-0.4 (-2.2, 1.5)	0.6964	+0.5 (0.1, 1.0)	0.0104
OpaD+	30	+0.3 (-1.9, 2.5)	0.7754	**+1.3 (0.5, 2.0)**	**0.0014**

Values in bold represent antigens where the % CD4^+^ T cell proliferation was statistically significant (p <0.01) compared to media alone or compared to the relevant Opa- antigen (REF). CI = confidence interval; PHA = phytohaemagglutinin; CEACAM = carcinoembryonic antigen-related cell adhesion molecule; SEB = Staphylococcal enterotoxin B; PBS = phosphate-buffered saline; LDAO = lauryldimethylamine-oxide; wt = wild-type.

### PBMCs with IL-2 only

**Fig 2:**
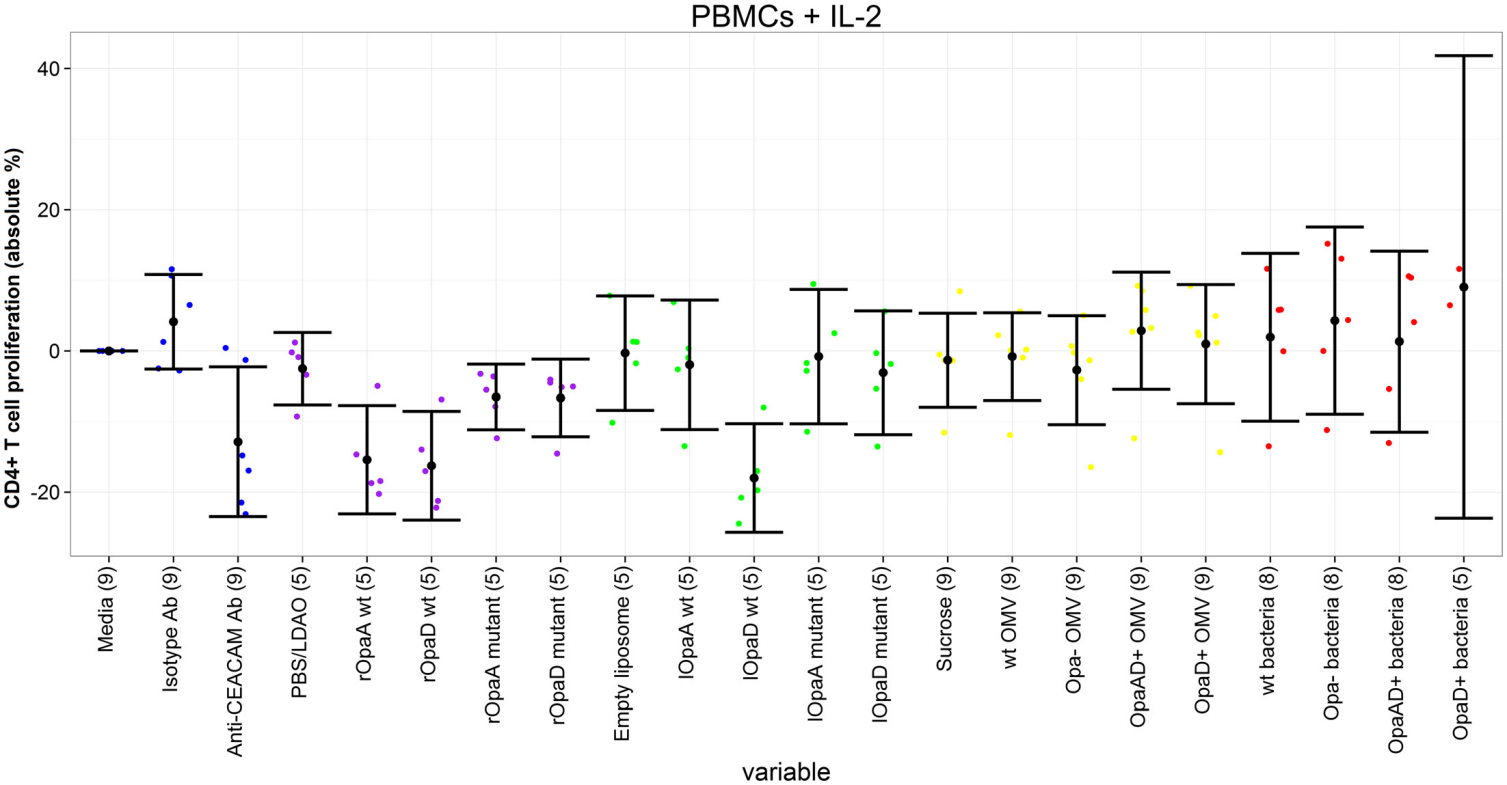
CD4+ T cell proliferation following incubation of PBMCs with different antigens, plus IL-2. Proliferation of each antigen relative to media only control. Each coloured dot represents the value for a single individual (control antigens—blue; recombinant proteins—purple, liposomal proteins—green, outer membrane vesicles—yellow, inactivated bacteria—red). Black dots represent mean value for each antigen, with 95% confidence intervals shown. Number in parentheses after each antigen represents number on individuals in that group. CEACAM = carcinoembryonic antigen-related cell adhesion molecule; SEB = Staphylococcal enterotoxin B; PBS = phosphate-buffered saline; LDAO = llauryldimethylamine-oxide; wt = wild-type. PHA (phytohaemagglutinin) result not shown for clarity, to confine y-axis to relevant range for other antigens.

A limited number of samples were tested with IL-2 only (up to 5 participants per antigen condition due to limited blood volumes available), to amplify responses by increasing activation and proliferation of T cells. In these conditions, all recombinant proteins caused inhibition of CD4^+^ T cell proliferation compared to media alone (between -16.3% and -6.5%), in addition to the liposomal wild-type OpaD (-18.0%) (Table 3, Fig 2). Wild-type recombinant Opa proteins were significantly more inhibitory than the mutated equivalents, with a difference of approximately 9% for OpaA and OpaD. Wild-type liposomal OpaD was also more inhibitory than the mutated form (difference 14.9%). None of the OMVs or inactivated bacteria were significantly stimulatory or inhibitory compared to the media only control, but Opa+ OMVs were slightly more stimulatory than Opa- OMVs. In contrast OpaA+ OpaD+ bacteria were less stimulatory than Opa- bacteria, although this difference was not observed with OpaD+ bacteria. All differences between Opa+ and Opa- OMVs and bacteria were relatively small in magnitude (between 3.0% and 5.6%).

**Table 3:** CD4^+^ T cell proliferation following incubation of PBMCs with different antigens, plus IL-2.

Antigen	Mean % CD4^+^ T-cell proliferation				
	n	vs. media (95% CI)	*p*-value	vs. Opa- (95% CI)	*p*-value
**Control antigens**					
PHA	5	**+53.5 (33.8, 73.3)**	**0.0009**	-	-
Isotype antibody	5	+4.1 (-2.6, 10.9)	0.1737	-	-
Anti-CEACAM antibody	5	-12.9 (-23.5, -2.3)	0.0263	-	-
**Recombinant proteins**					
Protein buffer (PBS/LDAO)	4	-2.5 (-7.6, 2.6)	0.2469	-	-
rOpaA wt	4	**-15.4 (-23.1, -7.7)**	**0.0051**	-8.9 (-16.0, -1.8)	0.0258
rOpaD wt	4	**-16.3 (-24.0, -8.5)**	**0.0043**	-9.6 (-16.6, -2.6)	0.0187
rOpaA mutant	4	-6.5 (-11.2, -1.9)	0.0176	REF	-
rOpaD mutant	4	-6.7 (-12.1, -1.2)	0.0282	REF	-
**Liposomal proteins**					
Empty liposome	4	-0.3 (-8.4, 7.8)	0.9250	-	-
lOpaA wt	4	-1.9 (-11.1, 7.2)	0.5872	-1.2 (-3.1, 0.7)	0.1641
lOpaD wt	4	**-18.0 (-25.7, -10.3)**	**0.0029**	-14.9 (-24.6, -5.2)	0.0129
lOpaA mutant	4	-0.8 (-10.3, 8.7)	0.8303	REF	-
lOpaD mutant	4	-3.1 (-11.8, 5.7)	0.3845	REF	-
**Outer membrane vesicles (OMVs)**					
OMV buffer (3% sucrose)	5	-1.3 (-8.0, 5.4)	0.6373	-	-
wt OMV	5	-0.8 (-7.0, 5.4)	0.7571	+1.9 (-0.7, 4.5)	0.1179
Opa- OMV	5	-2.7 (-10.4, 5.0)	0.4073	REF	-
OpaA+ OpaD+ OMV	5	+2.9 (-5.4, 11.2)	0.4150	**+5.6 (2.9, 8.3)**	**0.0030**
OpaD+ OMV	5	+1.0 (-7.4, 9.4)	0.7751	**+3.7 (1.8, 5.6)**	**0.0040**
**Inactivated bacteria**					
wt H44/76	4	+2.0 (-9.9, 13.9)	0.6700	-2.3 (-7.5, 2.8)	0.2783
Opa-	4	+4.3 (-8.9, 17.5)	0.4178	REF	-
OpaA+ OpaD+	4	+1.3 (-11.5, 14.2)	0.7869	-3.0 (-5.6, -0.3)	0.0351
OpaD+	1	+9.1 (-23.7, 41.8)	0.1765	-5.1 (-51.4, 41.2)	0.3949

Values in bold represent antigens where the % CD4^+^ T cell proliferation was statistically significant (p <0.01) compared to media alone or compared to the relevant Opa- antigen (REF). CI = confidence interval; PHA = phytohaemagglutinin; CEACAM = carcinoembryonic antigen-related cell adhesion molecule; PBS = phosphate-buffered saline; LDAO = llauryldimethylamine-oxide; wt = wild-type.

### PBMCs and purified CD4^+^ T cells with IL-2, anti-CD3 and anti-CD28

**Fig 3:**
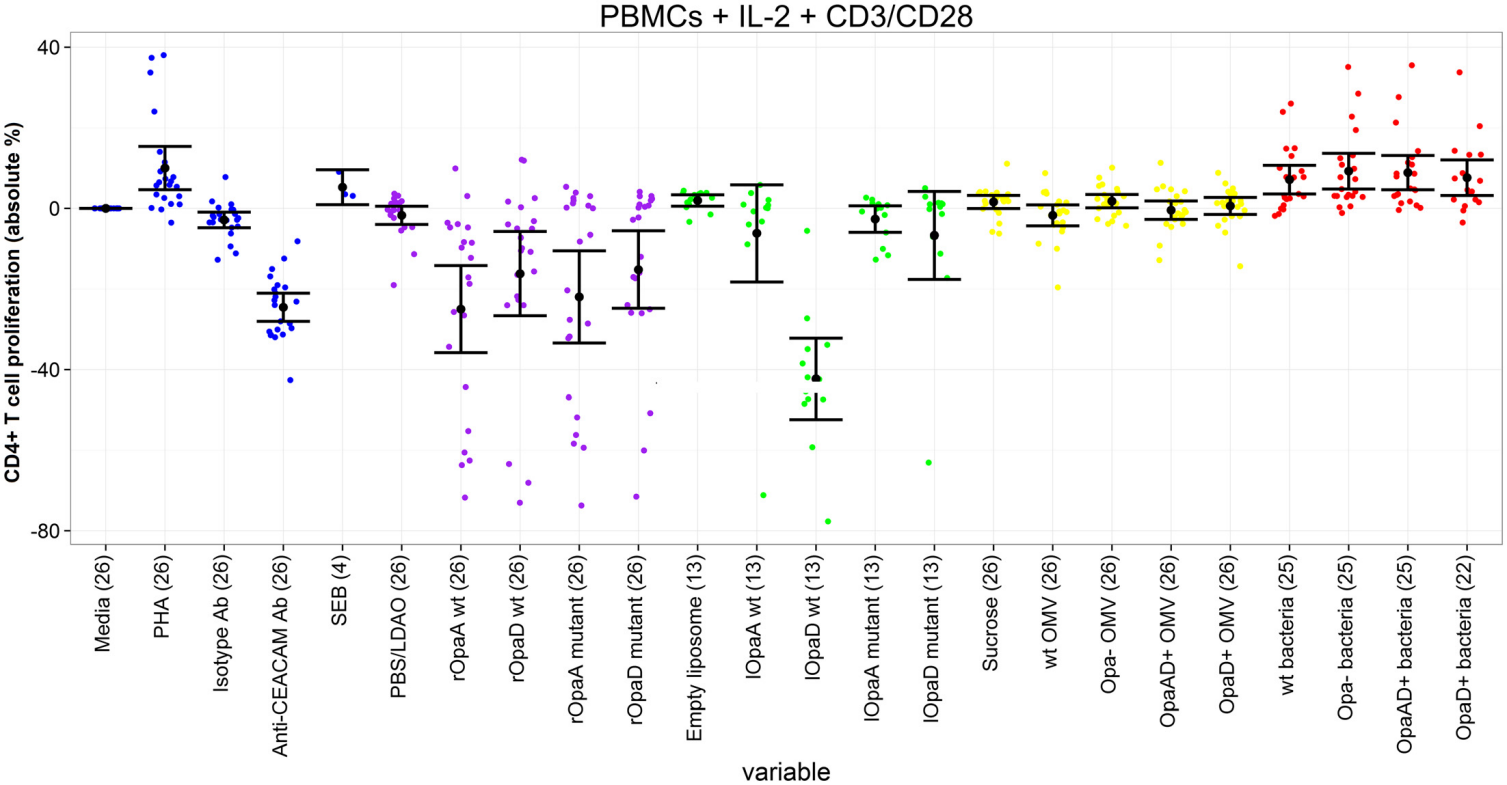
CD4^+^ T cell proliferation following incubation of PBMCs with different antigens, plus IL-2, anti-CD3 and anti-CD28. Proliferation of each antigen relative to media only control. Each coloured dot represents the value for a single individual (control antigens—blue; recombinant proteins—purple, liposomal proteins—green, outer membrane vesicles—yellow, inactivated bacteria—red). Black dots represent mean value for each antigen, with 95% confidence intervals shown. Number in parentheses after each antigen represents number on individuals in that group. PHA = phytohaemagglutinin; CEACAM = carcinoembryonic antigen-related cell adhesion molecule; SEB = Staphylococcal enterotoxin B; PBS = phosphate-buffered saline; LDAO = llauryldimethylamine-oxide; wt = wild-type.

**Fig 4:**
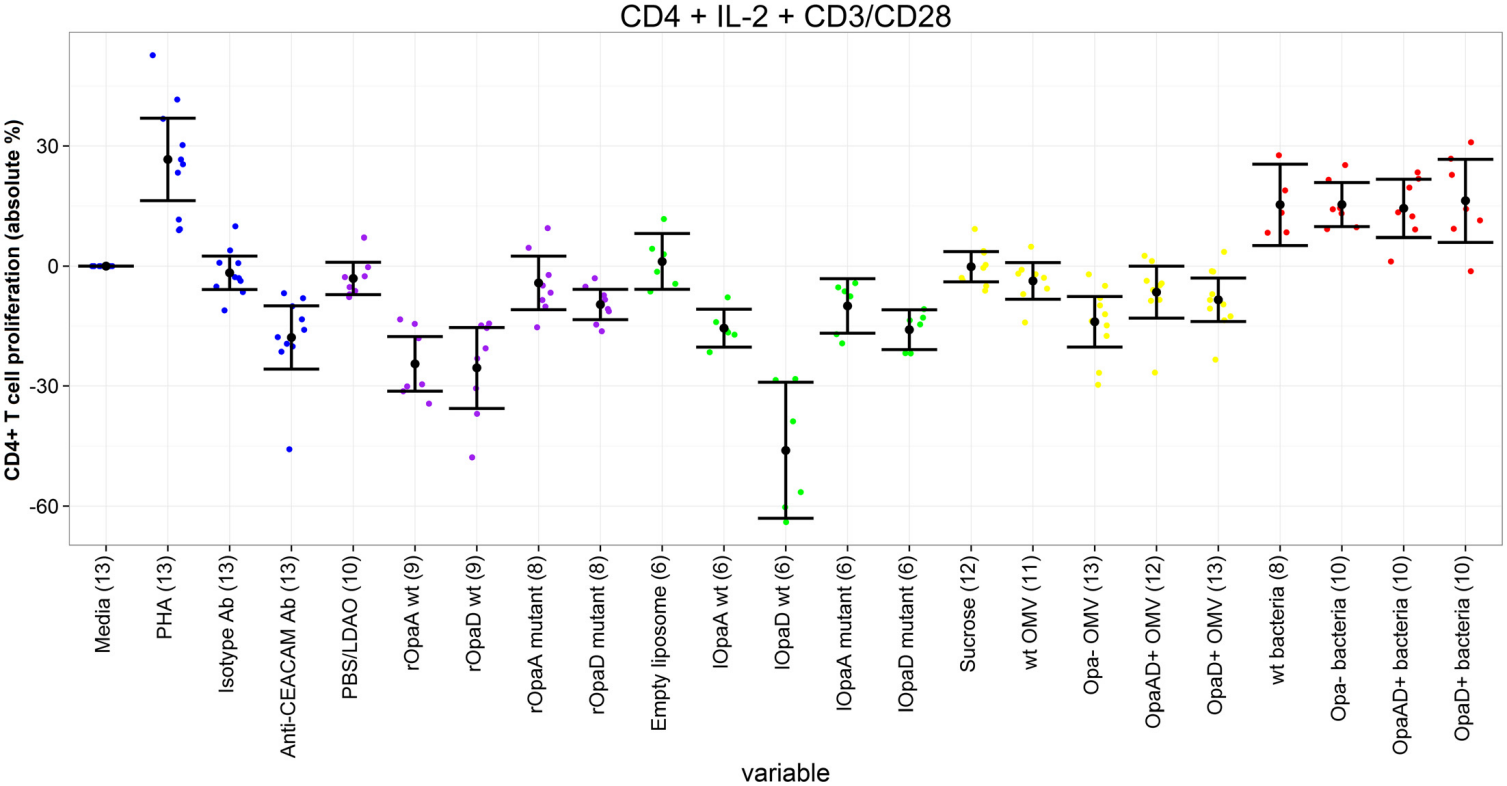
CD4^+^ T cell proliferation following incubation of purified CD4^+^ T cells with different antigens, plus IL-2, anti-CD3 and anti-CD28. Proliferation of each antigen relative to media only control. Each coloured dot represents the value for a single individual (control antigens—blue; recombinant proteins—purple, liposomal proteins—green, outer membrane vesicles—yellow, inactivated bacteria—red). Black dots represent mean value for each antigen, with 95% confidence intervals shown. Number in parentheses after each antigen represents number on individuals in that group. PHA = phytohaemagglutinin; CEACAM = carcinoembryonic antigen-related cell adhesion molecule; SEB = Staphylococcal enterotoxin B; PBS = phosphate-buffered saline; LDAO = llauryldimethylamine-oxide; wt = wild-type.

In these culture conditions, CD4^+^ cells are highly stimulated non-specifically, so inhibitory effects would expect to be seen. When purified CD4^+^ T cells were used, there was significant inhibition of CD4^+^ proliferation by all recombinant proteins and liposomes, with the exception of mutated rOpaA (although the trend was also towards inhibition in this case). Inhibition of proliferation ranged between -9.6% (rOpaD mutant) and -46.1% (lOpaD wild-type) (Tables 4 and 5, Figs 3 and 4). The Opa+ recombinant and liposomal proteins were significantly more inhibitory than the Opa- forms (with the exception of liposomal OpaA which also showed the same trend), with differences of between 5.6% and 30.1%. In addition, there was inhibition of CD4^+^ T cell proliferation by all OMVs (reaching statistical significance for all except wild-type), but Opa+ OMVs were less inhibitory than Opa- OMVs. All of the bacterial preparations tested were able to provide additional stimulation to CD4^+^ T cells in these conditions, with no significant differences between Opa+ and Opa- bacteria.

**Table 4:** CD4^+^ T cell proliferation following incubation of PBMCs with different antigens, plus IL-2, anti-CD3 and anti-CD28.

Antigen	Mean % CD4^+^ T-cell proliferation				
	n	vs. media (95% CI)	*p*-value	vs. Opa- (95% CI)	*p*-value
**Control antigens**					
PHA	21	**+10.0 (4.6, 15.4)**	**0.0009**	-	-
Isotype antibody	21	**-2.9 (-4.8, -0.9)**	**0.0057**	-	-
Anti-CEACAM antibody	21	**-24.5 (-28.0, -21.0)**	**<0.0001**	-	-
SEB	3	+5.3 (0.9, 9.6)	0.0308	-	-
**Recombinant proteins**					
Protein buffer (PBS/LDAO)	21	-1.7 (-4.0, 0.5)	0.1272	-	-
rOpaA wt	21	**-25.0 (-35.8, -14.1)**	**0.0001**	-3.0 (-9.7, 3.6)	0.3552
rOpaD wt	21	**-16.2 (-26.6, -5.7)**	**0.0041**	-1.0 (-4.6, 2.6)	0.5634
rOpaA mutant	21	**-21.9 (-33.4, -10.5)**	**0.0007**	REF	-
rOpaD mutant	21	**-15.2 (-24.8, -5.6)**	**0.0035**	REF	-
**Liposomal proteins**					
Empty liposome	12	+2.0 (0.5, 3.4)	0.0106	-	-
lOpaA wt	12	-6.2 (-18.2, 5.9)	0.2848	-3.5 (-13.6, 6.5)	0.4584
lOpaD wt	12	**-42.3 (-52.4, -32.2)**	**<0.0001**	**-35.6 (-48.4, -22.8)**	**0.0001**
lOpaA mutant	12	-2.7 (-6.0, 0.6)	0.1042	REF	-
lOpaD mutant	12	-6.7 (-17.6, 4.2)	0.2064	REF	-
**Outer membrane vesicles (OMVs)**					
OMV buffer (3% sucrose)	21	+1.6 (0.0, 3.2)	0.0546	-	-
wt OMV	21	-1.7 (-4.3, 0.9)	0.1805	-3.5 (-6.3, -0.8)	0.0146
Opa- OMV	21	+1.8 (0.1, 3.5)	0.0353	REF	-
OpaA+ OpaD+ OMV	21	-0.4 (-2.7, 1.8)	0.6900	-2.2 (-4.5, 0.0)	0.0513
OpaD+ OMV	21	+0.6 (-1.5, 2.7)	0.5541	-1.2 (-2.7, 0.3)	0.1108
**Inactivated bacteria**					
wt H44/76	20	**+7.1 (3.6, 10.7)**	**0.0005**	**-2.1 (-3.4, -0.8)**	**0.0036**
Opa-	20	**+9.3 (4.8, 13.7)**	**0.0003**	REF	-
OpaA+ OpaD+	20	**+8.9 (4.6, 13.2)**	**0.0003**	-0.4 (-1.7, 1.0)	0.5831
OpaD+	17	**+7.6 (3.2, 12.1)**	**0.0020**	-2.8 (-4.9, 0.7)	0.0115

Values in bold represent antigens where the % CD4^+^ T cell proliferation was statistically significant (p <0.01) compared to media alone or compared to the relevant Opa- antigen (REF). CI = confidence interval; PHA = phytohaemagglutinin; CEACAM = carcinoembryonic antigen-related cell adhesion molecule; SEB = Staphylococcal enterotoxin B; PBS = phosphate-buffered saline; LDAO = llauryldimethylamine-oxide; wt = wild-type.

**Table 5:** CD4^+^ T cell proliferation following incubation of purified CD4^+^ T cells with different antigens, plus IL-2, anti-CD3 and anti-CD28.

Antigen	Mean % CD4^+^ T-cell proliferation				
	n	vs. media (95% CI)	*p*-value	vs. Opa- (95% CI)	*p*-value
**Control antigens**					
PHA	9	**+26.6 (16.3, 37.0)**	**0.0002**	-	-
Isotype antibody	9	-1.7 (-5.9, 2.5)	0.3891	-	-
Anti-CEACAM antibody	9	**-17.9 (-25.8, -9.9)**	**0.0006**	-	-
**Recombinant proteins**					
Protein buffer (PBS/LDAO)	7	-3.1 (-7.1, 1.0)	0.1148	-	-
rOpaA wt	7	**-24.5 (-31.3, -17.6)**	**0.0001**	**-20.3 (-27.7, -12.8)**	**0.0004**
rOpaD wt	7	**-25.5 (-35.6, -15.4)**	**0.0006**	-15.9 (-28.1, -3.6)	0.0183
rOpaA mutant	7	-4.2 (-10.9, 2.5)	0.1818	REF	-
rOpaD mutant	7	**-9.6 (-13.4, -5.8)**	**0.0005**	REF	-
**Liposomal proteins**					
Empty liposome	5	+1.1 (-5.8, 8.1)	0.6903	-	-
lOpaA wt	5	**-15.5 (-20.3, -10.8)**	**0.0004**	-5.6 (-11.2, 0.0)	0.0512
lOpaD wt	5	**-46.1 (-63.1, -29.1)**	**0.0009**	-30.1 (-51.6, -8.6)	0.0155
lOpaA mutant	5	-10.0 (-16.8, -3.1)	0.0132	REF	-
lOpaD mutant	5	**-15.9 (-20.9, -10.9)**	**0.0004**	REF	-
**Outer membrane vesicles (OMVs)**					
OMV buffer (3% sucrose)	8	-0.1 (-3.9, 3.6)	0.9296	-	-
wt OMV	7	-3.7 (-8.3, 0.9)	0.0979	**+11.0 (4.7, 17.3)**	**0.0046**
Opa- OMV	9	**-13.9 (-20.3, -7.6)**	**0.0008**	REF	-
OpaA+ OpaD+ OMV	8	-6.5 (-13.0, 0.0)	0.0497	**+8.1 (2.7, 13.5)**	**0.0087**
OpaD+ OMV	9	**-8.4 (-13.9, -3.0)**	**0.0067**	+5.5 (1.5, 9.5)	0.0120
**Inactivated bacteria**					
wt H44/76	4	+15.3 (5.2, 25.4)	0.0139	-1.4 (-4.7, 1.8)	0.2909
Opa-	6	**+15.3 (9.8, 20.8)**	**0.0005**	REF	-
OpaA+ OpaD+	6	**+14.4 (7.1, 21.7)**	**0.0029**	-0.9 (-7.4, 5.5)	0.7349
OpaD+	6	**+16.3 (5.9, 26.7)**	**0.0085**	+1.0 (-6.8, 8.7)	0.7701

Values in bold represent antigens where the % CD4^+^ T cell proliferation was statistically significant (p <0.01) compared to media alone or compared to the relevant Opa- antigen (REF). CI = confidence interval; PHA = phytohaemagglutinin; CEACAM = carcinoembryonic antigen-related cell adhesion molecule; PBS = phosphate-buffered saline; LDAO = llauryldimethylamine-oxide; wt = wild-type.

When mixed PBMCs were used in the same highly stimulated culture conditions, results for the recombinant and liposomal proteins were similar to those found using purified CD4^+^ T cells, with inhibition observed for all preparations, although fewer achieved statistical significance. On comparison of the mutated and wild-type proteins there was only a significant difference for liposomal OpaD, with the wild-type protein again being more inhibitory than the mutated protein (difference 35.6%). All of the bacterial strains tested were able to provide additional stimulation to CD4^+^ T cells, as observed with purified CD4^+^ T cells, and wild-type and OpaD+ bacteria were slightly less stimulatory than Opa- bacteria. Most OMVs in these conditions were not significantly stimulatory or inhibitory, although a slight stimulatory effect was observed with Opa- OMVs.

A comparison of results from this study and previous studies of Opa-mediated effects on CD4^+^ T cells is summarised in Table 6.

**Table 6:** Studies of the effects of Opa proteins on CD4^+^ T cell proliferation.

Subjects	Antigens	Culture conditions	Method of assessment	Results	Ref
18 healthy adults (29–55 years)	Purified NM Opa proteins (3 variants)	PBMCs *ex vivo*	[^3^H]thymidine incorporation	Opa induced proliferation in most donors, greater than Opc or PorA	[16]
≥4 individuals per experiment, no further details	Live NG expressing different Opa variants (strain MS11)	Purified CD4^+^ T cells* +/- IL-2 (48 hours pre-incubation) +/- anti-CD3 +/- anti-CD28	Lymphocyte density, assessed by direct counting	HSPG-binding Opa^+^ bacteria induced greater proliferation than CEACAM-binding Opa^+^ bacteria	[31]
Not stated	OMVs from Opa^+^ NM (strain K454) and Opa^-^ NL (strain Y92 1009)	Purified CD4^+^ T cells + IL-2 (48 hours pre-incubation) + anti-CD3 + anti-CD28	Cell culture density, assessed by direct counting	NL Opa^-^ OMVs induced greater proliferation than NM Opa^+^ OMVs	[24]
	OMVs from NG expressing different Opa variants (strain MS11)	Jurkat CD4^+^ T cells† +/- IL-2 (48h) and/or anti-CD3		Opa^-^ or HSPG-binding Opa^+^ OMVs induced greater proliferation than CEACAM-binding Opa^+^ OMVs	
Healthy adults and buffy coats, number not stated	Live Opa^+^ or Opa^-^ NM (strain C751 or H44/76)	Purified CD4^+^ T cells‡ +/- IL-2 (48 hours pre-incubation) +/- anti-CD3	[^3^H]thymidine incorporation	No difference in proliferation between isogenic Opa^+^ and Opa^-^ strains	[18]
	Live Opa^+^ or Opa^-^ NM (strain C751) **or** heat-killed Opa^+^ or Opa^-^ NM (strain H44/76) **or** Opa^+^ or Opa^-^ NM OMVs (strain H44/76)	Purified CD4^+^ T cells + IL-2 (96 hours pre-incubation) +/- anti-CD3 and anti-CD28	Flow cytometric analysis of CFSE dilution	No difference in proliferation between isogenic Opa^+^ and Opa^-^ strains	
46 healthy adults (18–66 years)	Wild-type and mutant Opa proteins, presented as recombinant proteins and liposome	PBMCs +/- IL-2 (48 hours) +/- anti-CD3 and anti-CD28 **or** purified CD4^+^ T cells + anti-CD3 and anti-CD28	Flow cytometric analysis of CFSE dilution	Proteins and liposomes generally inhibitory in most conditions, with wt variants more inhibitory than mutants	this study
	Opa^+^ or Opa^-^ NM OMVs **or** ethanol-fixed Opa^+^ or Opa^-^ NM (strain H44/76)			OMVs all generally inhibitory only when using purified CD4^+^ T cells. Bacteria all stimulatory in the presence of anti-CD3 and anti-CD28. No consistent difference between Opa^+^ and Opa^-^ OMVs or bacteria	

NM = *N*. *meningitidis*, NG = *N*. *gonorrhoeae*, NL = *N*. *lactamica*; OMV = outer membrane vesicle; HSPG = heparin sulphate proteoglycan; CEACAM = carcinoembryonic antigen-related cell adhesion molecule;

*conditions included CD4^+^ T cells alone, with IL-2 or anti-CD3 only, with IL-2 and anti-CD3, with anti-CD3 and anti-CD28, and with IL-2, anti-CD3 and anti-CD28;

^†^conditions included CD4^+^ T cells alone, with IL-2 or anti-CD3 only, and with IL-2 and anti-CD3;

^‡^conditions included CD4^+^ T cells alone, with anti-CD3 only, and with IL-2 and anti-CD3.

### Bactericidal antibody responses following immunisation of mice with recombinant Opa proteins

The immunogenicity of recombinant forms of wild-type and mutated OpaA and OpaD from H44/76 were tested using Opa+ and Opa- strains as target strains in the serum bactericidal antibody (SBA) assay. Data using wild type proteins has been reported previously, and are included here for comparison to the mutated forms [26]. The recombinant proteins resulted in titres of 1:256 when the target strain expressed the same Opa variant used for immunisation (Table 7), and <1:4 otherwise. Introduction of mutations in the hypervariable HV2 regions led to complete abrogation of SBA activity for OpaA, but had no effect on the SBA titre for OpaD against either OpaD+ target strain.

**Table 7:** Serum bactericidal antibody titres of pooled murine sera against 4 target strains, following immunisation with wild-type and mutant recombinant OpaA and OpaD proteins.

	Target strain in SBA assay (Opa phenotype)			
Recombinant protein used for immunisation	H44/76 (Opa-)	M014 (Opa-)	M002 (OpaD+)	M001 (OpaA+ OpaD+)
OpaA wt	<1:4	<1:4	<1:4	1:256
OpaD wt	<1:4	<1:4	1:256	1:256
OpaA mutant	<1:4	<1:4	<1:4	<1:4
OpaD mutant	<1:4	<1:4	1:256	1:256

Titres represent highest dilution at which there was 50% bacterial survival. wt = wild-type.

## Discussion

In this study, a range of Opa+ and Opa- antigens was used in culture with both PBMCs and purified CD4^+^ T cells to demonstrate that recombinant and, to a lesser extent, liposomal OpaA and OpaD proteins from *N*. *meningitidis* strain H44/76 inhibited CD4^+^ T cells *in vitro* when cells were pre-stimulated with IL-2, with or without anti-CD3 and anti-CD28 antibodies. When the HV2 region of these Opa proteins was mutated to abrogate CEACAM1 binding, these inhibitory effects were usually reduced, although inhibition still occurred. These same mutations had variable effects on immunogenicity of these proteins when used for immunisation in mice—bactericidal antibodies were still induced with OpaD, but not for OpaA. It is not possible to directly contrast the T cell studies with mouse immunisations because of the different CEACAM repertoire found in humans and mice. In contrast, OMVs and inactivated bacteria based on Opa+ and Opa- strains did not have the same inhibitory effects on CD4^+^ T cells. All bacterial strains were typically stimulatory and there were no consistent differences between Opa+ and Opa- bacteria or OMVs. It is likely, therefore, that other proteins contained within these preparations are immunodominant with respect to these *in vitro* interactions.

This study was the first to study the culture of freshly isolated PBMCs *ex vivo* to assess Opa-mediated interactions, and under these conditions none of the Opa+ or Opa- antigens exerted a major stimulatory or inhibitory effect on CD4^+^ T cells. This is a standard method of assessing *in vitro* T cell responses to vaccine candidates and the assessment of antigen-specific proliferation [35] following vaccination. In this study, T cell responses were assessed in healthy adults without recent meningococcal vaccination or infection, which may explain the relative lack of proliferative responses observed in the PBMC *ex vivo* conditions. This study, therefore, does not support the argument that Opa proteins would have an inhibitory effect *in vivo* if given as a vaccine to healthy adults. Immune responses to previous OMV vaccines have included the induction of anti-Opa antibodies, which also suggests that Opa does not have a significant immunosuppressive effect when included within OMVs.

Previously published data regarding the effects of meningococcal Opa proteins on CD4^+^ T cell proliferation have been conflicting, creating a significant hurdle in the development of any Opa-containing vaccines (Table 6). Studies comparing the meningococcal proteins PorA, PorB, Opa and Opc showed that Opa induced the strongest T cell proliferative responses [16, 17] and whole bacteria and OMVs have been shown to stimulate CD4^+^ T cell proliferation, independent of their Opa phenotype [18]. Several Opa-containing OMV vaccines are immunogenic in humans [19–23]. In other studies, when CD4^+^ T cells were exposed to OMVs from Opa-expressing meningococci or to gonococci expressing CEACAM-binding Opa variants, their activation and proliferation in response to a variety of stimuli were effectively halted [24, 31].

There may be a number of reasons to explain the differences between various studies. Different studies have used different Opa variants and Opa proteins from H44/76 have not previously been shown to have an inhibitory effect. The proposed mechanism of inhibition is via an Opa-CEACAM1 interaction, and both Opa proteins used in this study bind to CEACAM1 [28]. When OMVs and bacteria are used, there may be variation of Opa expression levels relative to other outer membrane proteins, resulting in different effects. Further major differences between studies are the culture conditions and methods of assessment of proliferation. PBMCs and purified CD4^+^ T cells have been used, with most studies including assays with IL-2 pre-incubation and/or addition of anti-CD3 and anti-CD28. Differences in duration of culture may also affect CEACAM1 expression on the T cell surface. While these conditions are useful in exploring specific interactions, they are unlikely to represent what occurs *in vivo*, and should be interpreted with caution when predicting the potential effects of Opa proteins during meningococcal infection or following immunisation in humans. IL-2 is a T cell growth factor and increases CEACAM1 expression, potentially enhancing any CEACAM1-mediated Opa-induced effects [31], supported by the inhibition of proliferation observed with the anti-CEACAM antibody in these conditions. IL-2 is produced *in vivo* during T cell responses, and it is likely that the PBMC *ex vivo* assay represents more physiological levels of IL-2, compared to those assays when exogenous IL-2 was added. Anti-CD3 and anti-CD28 antibodies provide signals for T cell activation, as well as further increasing CEACAM1 expression on the T cell surface [31]. Incubation of cells with IL-2, anti-CD3 and anti-CD28 therefore tends to lead to inhibitory rather than stimulatory effects. The greater baseline stimulation of cells where anti-CD3 and anti-CD28 have been added is also the most likely explanation for the greater amplitude of inhibition observed in these conditions, compared to with IL-2 alone. The addition of PHA in the presence of anti-CD3 and anti-CD28 resulted in less additional proliferation than when they were absent, confirming these cells were already highly stimulated. Methods of measuring CD4^+^ T cell proliferation have varied between each of the published studies. A flow cytometry approach based on CFSE dilution was adopted in this study to ensure that dead cells were excluded from the analysis, but this is more difficult to achieve when using other methods described.

Our data support the conclusion that isolated Opa proteins have a different effect to Opa expressed on the bacterial surface with other proteins. Considering that *Neisseria* have the capacity to randomly and reversibly switch many surface antigens ‘on’ and ‘off’ by a process of PV, it is also possible that the differential expression of uncharacterised factors other than Opa may influence T cell responses during infection. However, we used OMVs and bacteria from isogenic strains, constructed from the same parent strain, to reduce this risk. We have previously shown that expression of the major proteins in these strains is the same [26]. The results from these experiments do not support a proposed hypothesis that Opa proteins would inhibit CD4^+^ T cell proliferation when part of whole bacteria (as would be the case during infection) or contained within an OMV vaccine. Furthermore, there was no consistent effect between Opa+ and Opa- OMVs or bacteria, suggesting that the effects of other proteins within these preparations was immunodominant over any Opa effect. This is further supported by a recent study showing no difference in SBA titres following immunisation of CEACAM1-transgenic mice with Opa+ and Opa- OMVs [36]. However, our study and the mouse immunisation study used strain H44/76 and it may be that different Opa proteins have different effects, highlighted by the differences observed between liposomal and recombinant OpaA and OpaD.

One major advantage of this study was the inclusion of a large number of healthy adult subjects. Most previous studies have not stated sample size and the use of buffy coats from the National Blood Service in one study [18] would make it impossible to have specific inclusion and exclusion criteria. Comparison of Opa+ and Opa- strains to determine Opa-dependent effects is only valid when comparing otherwise isogenic strains, as used in this study. Although most previous studies have also used isogenic strains, one study compared Opa+ OMVs from *N*. *meningitidis* with Opa- OMVs from *N*. *lactamica* where many other surface proteins would also be different and may exert an immunomodulatory effect.

This is the first study of the effects of Opa proteins on CD4^+^ T cells to include a range of culture conditions, including the use of PBMCs and purified CD4^+^ T cells, using the same set of antigens with the same measure of proliferation. Different types of Opa+ and Opa- antigens were included to detect a consistent effect of Opa on CD4^+^ T cells. The lack of inhibitory responses observed with PBMCs *ex vivo* suggest that Opa proteins would not cause immunosuppressive effects if included as a vaccine component. Opa proteins inhibited CD4^+^ T cell proliferation after stimulation with IL-2, anti-CD3 and anti-CD28, possibly due to upregulation of CEACAM1 on the T cell surface and/or greater baseline stimulation, which would tend to highlight any inhibitory effects. Future studies would be required to dissect which CD4^+^ subpopulation is responsible for the effects observed. Opa proteins alone inhibited T cell responses in pre-stimulated *in vitro* conditions—an effect which was reduced by mutation of the CEACAM1-binding regions. However, when included with other antigens in the form of OMVs or killed bacteria, no Opa-dependent inhibition was observed, providing reassurance that current or future meningococcal vaccines containing OMVs would not be negatively affected by Opa protein expression.

## References

[1] World Health Organization. Control of epidemic meningococcal disease WHO practical guidelines. 2nd edition Geneva, Switzerland: World Health Organization, 1998.

[2] Gray SJ, Trotter CL, Ramsay ME, Guiver M, Fox AJ, Borrow R, et al Epidemiology of meningococcal disease in England and Wales 1993/94 to 2003/04: contribution and experiences of the Meningococcal Reference Unit. J Med Microbiol. 2006;55(Pt 7):887–96. . 16772416 10.1099/jmm.0.46288-0

[3] Ramsay M, Fox A. EU-IBIS Network. Invasive Neisseria meningitidis in Europe 2006. London: Health Protection Agency, 2007.

[4] Baethgen LF, Weidlich L, Moraes C, Klein C, Nunes LS, Cafrune PI, et al Epidemiology of meningococcal disease in southern Brazil from 1995 to 2003, and molecular characterization of Neisseria meningitidis using multilocus sequence typing. Trop Med Int Health. 2008;13(1):31–40. 10.1111/j.1365-3156.2007.01970.x 18290999

[5] Rosenstein NE, Perkins BA, Stephens DS, Popovic T, Hughes JM. Meningococcal disease. N Engl J Med. 2001;344(18):1378–88. . 11333996 10.1056/NEJM200105033441807

[6] Baker MG, Martin DR, Kieft CE, Lennon D. A 10-year serogroup B meningococcal disease epidemic in New Zealand: descriptive epidemiology, 1991–2000. J Paediatr Child Health. 2001;37(5):S13–9. . 11885731 10.1046/j.1440-1754.2001.00722.x

[7] Parkhill J, Achtman M, James KD, Bentley SD, Churcher C, Klee SR, et al Complete DNA sequence of a serogroup A strain of Neisseria meningitidis Z2491. Nature. 2000;404(6777):502–6. . 10761919 10.1038/35006655

[8] Tettelin H, Saunders NJ, Heidelberg J, Jeffries AC, Nelson KE, Eisen JA, et al Complete genome sequence of Neisseria meningitidis serogroup B strain MC58. Science. 2000;287(5459):1809–15. . 10710307 10.1126/science.287.5459.1809

[9] Bhat KS, Gibbs CP, Barrera O, Morrison SG, Jahnig F, Stern A, et al The opacity proteins of Neisseria gonorrhoeae strain MS11 are encoded by a family of 11 complete genes. Mol Microbiol. 1991;5(8):1889–901. . 1815562 10.1111/j.1365-2958.1991.tb00813.x

[10] Gray-Owen SD. Neisserial Opa proteins: impact on colonization, dissemination and immunity. Scand J Infect Dis. 2003;35(9):614–8. . 14620144 10.1080/00365540310016042

[11] Virji M, Makepeace K, Ferguson DJ, Achtman M, Moxon ER. Meningococcal Opa and Opc proteins: their role in colonization and invasion of human epithelial and endothelial cells. Mol Microbiol. 1993;10(3):499–510. . 7968528 10.1111/j.1365-2958.1993.tb00922.x

[12] Sadarangani M, Pollard AJ, Gray-Owen SD. Opa proteins and CEACAMs: pathways of immune engagement for pathogenic Neisseria. FEMS Microbiol Rev. 2011;35(3):498–514. 10.1111/j.1574-6976.2010.00260.x . 21204865

[13] Callaghan MJ, Lewis S, Sadarangani M, Bailey SE, Chan H, Ferguson DJ, et al The potential of recombinant Opa proteins as vaccine candidates against hyperinvasive meningococci. Infect Immun. 2011 Epub 2011/04/06. IAI.01338-10 [pii] 10.1128/IAI.01338-10 . 21464082 PMC3191958

[14] Hedges SR, Mayo MS, Mestecky J, Hook EW 3rd, Russell MW. Limited local and systemic antibody responses to Neisseria gonorrhoeae during uncomplicated genital infections. Infect Immun. 1999;67(8):3937–46. . 10417159 10.1128/iai.67.8.3937-3946.1999 PMC96675

[15] Pollard AJ, Galassini R, Rouppe van der Voort EM, Hibberd M, Booy R, Langford P, et al Cellular immune responses to Neisseria meningitidis in children. Infect Immun. 1999;67(5):2452–63. Epub 1999/05/04. 10225908 10.1128/iai.67.5.2452-2463.1999 PMC115991

[16] Wiertz EJ, Delvig A, Donders EM, Brugghe HF, van Unen LM, Timmermans HA, et al T-cell responses to outer membrane proteins of Neisseria meningitidis: comparative study of the Opa, Opc, and PorA proteins. Infect Immun. 1996;64(1):298–304. . 8557355 10.1128/iai.64.1.298-304.1996 PMC173759

[17] Wiertz EJ, van Gaans-van den Brink JA, Schreuder GM, Termijtelen AA, Hoogerhout P, Poolman JT. T cell recognition of Neisseria meningitidis class 1 outer membrane proteins. Identification of T cell epitopes with selected synthetic peptides and determination of HLA restriction elements. J Immunol. 1991;147(6):2012–8. . 1716291

[18] Youssef AR, van der Flier M, Estevao S, Hartwig NG, van der Ley P, Virji M. Opa+ and Opa- isolates of Neisseria meningitidis and Neisseria gonorrhoeae induce sustained proliferative responses in human CD4+ T cells. Infect Immun. 2009;77(11):5170–80. 10.1128/IAI.00355-09 19720754 PMC2772550

[19] Bjune G, Hoiby EA, Gronnesby JK, Arnesen O, Fredriksen JH, Halstensen A, et al Effect of outer membrane vesicle vaccine against group B meningococcal disease in Norway. Lancet. 1991;338(8775):1093–6. . 1682541 10.1016/0140-6736(91)91961-s

[20] Sierra GV, Campa HC, Varcacel NM, Garcia IL, Izquierdo PL, Sotolongo PF, et al Vaccine against group B Neisseria meningitidis: protection trial and mass vaccination results in Cuba. NIPH Ann. 1991;14(2):195–207; discussion 8–10. . 1812432

[21] de Moraes JC, Perkins BA, Camargo MC, Hidalgo NT, Barbosa HA, Sacchi CT, et al Protective efficacy of a serogroup B meningococcal vaccine in Sao Paulo, Brazil. Lancet. 1992;340(8827):1074–8. . 1357461 10.1016/0140-6736(92)93086-3

[22] Noronha CP, Struchiner CJ, Halloran ME. Assessment of the direct effectiveness of BC meningococcal vaccine in Rio de Janeiro, Brazil: a case-control study. Int J Epidemiol. 1995;24(5):1050–7. . 8557439 10.1093/ije/24.5.1050

[23] Oster P, Lennon D, O'Hallahan J, Mulholland K, Reid S, Martin D. MeNZB: a safe and highly immunogenic tailor-made vaccine against the New Zealand Neisseria meningitidis serogroup B disease epidemic strain. Vaccine. 2005;23(17–18):2191–6. . 15755593 10.1016/j.vaccine.2005.01.063

[24] Lee HS, Boulton IC, Reddin K, Wong H, Halliwell D, Mandelboim O, et al Neisserial outer membrane vesicles bind the coinhibitory receptor carcinoembryonic antigen-related cellular adhesion molecule 1 and suppress CD4+ T lymphocyte function. Infect Immun. 2007;75(9):4449–55. . 17620353 10.1128/IAI.00222-07 PMC1951172

[25] Lyons AB, Hasbold J, Hodgkin PD. Flow cytometric analysis of cell division history using dilution of carboxyfluorescein diacetate succinimidyl ester, a stably integrated fluorescent probe. Methods Cell Biol. 2001;63:375–98. Epub 2000/11/04. . 11060850 10.1016/s0091-679x(01)63021-8

[26] Sadarangani M, Hoe JC, Callaghan MJ, Jones C, Chan H, Makepeace K, et al Construction of Opa-positive and Opa-negative strains of Neisseria meningitidis to evaluate a novel meningococcal vaccine. PLoS One. 2012;7(12):e51045 Epub 2012/12/20. 10.1371/journal.pone.0051045 PONE-D-12-14740 [pii]. 23251421 PMC3521020

[27] Arigita C, Kersten GF, Hazendonk T, Hennink WE, Crommelin DJ, Jiskoot W. Restored functional immunogenicity of purified meningococcal PorA by incorporation into liposomes. Vaccine. 2003;21(9–10):950–60. Epub 2003/01/28. S0264410X02005467 [pii]. . 12547608 10.1016/s0264-410x(02)00546-7

[28] de Jonge MI, Hamstra HJ, van Alphen L, Dankert J, van der Ley P. Mapping the binding domains on meningococcal Opa proteins for CEACAM1 and CEA receptors. Mol Microbiol. 2003;50(3):1005–15. . 14617157 10.1046/j.1365-2958.2003.03749.x

[29] Chilson OP, Kelly-Chilson AE. Mitogenic lectins bind to the antigen receptor on human lymphocytes. Eur J Immunol. 1989;19(2):389–96. Epub 1989/02/01. 10.1002/eji.1830190225 . 2703017

[30] Kay JE. Mechanisms of T lymphocyte activation. Immunol Lett. 1991;29(1–2):51–4. Epub 1991/07/01. . 1916924 10.1016/0165-2478(91)90198-j

[31] Boulton IC, Gray-Owen SD. Neisserial binding to CEACAM1 arrests the activation and proliferation of CD4+ T lymphocytes. Nature immunology. 2002;3(3):229–36. . 11850628 10.1038/ni769

[32] Borrow R, Carlone GM. Serogroup B and C Serum Bactericidal Assays In: Pollard AJ, Maiden MC, editors. Meningococcal Vaccines Methods and Protocols. Totowa, New Jersey: Humana Press Inc.; 2001 p. 289–304. 10.1385/1-59259-148-5:289 21336762

[33] Plebanski M, Burtles SS. In vitro primary responses of human T cells to soluble protein antigens. J Immunol Methods. 1994;170(1):15–25. Epub 1994/03/29. . 7512606 10.1016/0022-1759(94)90241-0

[34] Kennell AS, Gould KG, Salaman MR. Proliferation assay amplification by IL-2 in model primary and recall antigen systems. BMC Res Notes. 2014;7:662 Epub 2014/09/23. 1756-0500-7-662 [pii] 10.1186/1756-0500-7-662 25239080 PMC4190572

[35] Dorrell L, Yang H, Ondondo B, Dong T, di Gleria K, Suttill A, et al Expansion and diversification of virus-specific T cells following immunization of human immunodeficiency virus type 1 (HIV-1)-infected individuals with a recombinant modified vaccinia virus Ankara/HIV-1 Gag vaccine. J Virol. 2006;80(10):4705–16. Epub 2006/04/28. 80/10/4705 [pii] 10.1128/JVI.80.10.4705-4716.2006 16641264 PMC1472080

[36] Zariri A, van Dijken H, Hamstra HJ, van der Flier M, Vidarsson G, van Putten JP, et al Expression of human CEACAM1 in transgenic mice limits the Opa-specific immune response against meningococcal outer membrane vesicles. Vaccine. 2013;31(47):5585–93. Epub 2013/08/13. S0264-410X(13)01055-4 [pii] 10.1016/j.vaccine.2013.07.069 . 23933369

